# Muscle Sensor Model Using Small Scale Optical Device for Pattern Recognitions

**DOI:** 10.1155/2013/346047

**Published:** 2013-10-10

**Authors:** Kreangsak Tamee, Khomyuth Chaiwong, Kriengsak Yothapakdee, Preecha P. Yupapin

**Affiliations:** ^1^Department of Computer Science and Information Technology, Faculty of Science, Naresuan University, Phitsanulok 65000, Thailand; ^2^Advanced Studies Center, Department of Physics, Faculty of Science, King Mongkut's Institute of Technology Ladkrabang, Bangkok 10520, Thailand; ^3^South East Asian Theoretical Physics Association (SEATPA), 50 Nanyang Avenue, Singapore 639798

## Abstract

A new sensor system for measuring contraction and relaxation of muscles by using a PANDA ring resonator is proposed. The small scale optical device is designed and configured to perform the coupling effects between the changes in optical device phase shift and human facial muscle movement, which can be used to form the relationship between optical phase shift and muscle movement. By using the Optiwave and MATLAB programs, the results obtained have shown that the measurement of the contraction and relaxation of muscles can be obtained after the muscle movements, in which the unique pattern of individual muscle movement from facial expression can be established. The obtained simulation results, that is, interference signal patterns, can be used to form the various pattern recognitions, which are useful for the human machine interface and the human computer interface application and discussed in detail.

## 1. Introduction

Pattern recognitions have been widely investigated and used in many areas of applications [1–6]. In recent years, researchers have attempted to analyze and research for disability [7–10], where most of them have built the system to assist or facilitate the ability. For instance, application was developed in order to use it in the work of the interaction between humans and computer or humans and machines, in which the human computer interface (HCI) is a method for controlling and data transmission where humans need to interact with the computer, such as in useful identify contraction and relaxation of facial muscles during speech and investigated hand gestures for HCI applications [11]. The human machine interface (HMI) systems have been used in applications with other devices such as controlling a virtual wheelchair through the hands-free control system based on multichannel forehead biosignals electromyography (EMG) which was suggested [12], especially, the human body surface, where the face is the most important area, in which all of the organs on the face could use to sense or present the message and to exchange the information with the real world [13]. Till date, the characteristics of the contraction and relaxation of the muscles of the face have become an excellent platform for developing sensor and pattern recognition, such as facial expression recognition (FER) in order to extract the emotional features [14], where the problem of recognizing the dynamic image plane has been described and solved by means of VTB and moments on spatiotemporal plane [15]. However, the problem remains; when all of these devices and interfacing methods are not suitable for those who are not able to move both the upper and lower parts, therefore, the facial expression is the only way that they could use to communicate [16]. At present, the searching of new devices and techniques for pattern recognitions remains. Recently, Yupapin and Sarapat have shown the interesting system that uses the small scale optical device known as a PANDA ring circuit [17], where the required parameters of the change device phase shift and muscle movement can be measured.

Optical devices and methods have been implemented and widely used in various applications, for instance, in communication [18], security [19], and agricultural [20] and medical applications [21–23], where one of the interesting works is in medical application. The use of optical devices has shown the good potentials for medical applications, where the medical diagnosis and ways to treat diseases such as the detection of human vital signs used health monitoring technology based on fiber optic interferometry to detect changes in the submicron length of the optical fiber [24], measurement of tissue hemodynamics and monitoring new tissue diagnostics [25], and using optical mammography for the detection of early stage breast cancer [26]. In operation, the optical device acts as an optical sensor to measure and monitor changes of wavelength that led to its refractive index change and optical path length, which can be used to form the required measurement parameters. In principle, the measurement principle of sensor is based on stimulations to the morphology of a dielectric microsphere due to electrostriction effect [27], which is a reaction caused by the external environment. The advantage of optical technique is the accuracy and precision on microscale or nanoscale of the applied sensors, which needs the high efficiency tools as well, where the integrated nonlinear optical PANDA type ring resonator has shown the potential of use for such requirements [28].

In this paper, a new sensor system using a small scale optical device for measuring contraction and relaxation of muscles has been designed and simulated for facial muscle movements. A nonlinear optical ring resonator known as a PANDA ring circuit has been proposed as the basic sensing device [29, 30], in which the form of sensor can be a thin film probe. The device material is InGaAsP/InP, which can also be coated by Au or other conducting materials, for example, Ag, Cu, and so forth, for wider applications. In principle, the change in phase of optical device within the sensing system is coupled by muscle movement at the sensing location, which can be related to the required measurement parameters. The changes in optical phase can be stimulated by facial muscle movements, where the different movements can occur and be measured. Finally, the specific movements can be configured by the interference fringes. The obtained interference fringes can be used to form the relationship between facial muscle movement and pattern recognitions, which is useful for computer machine language and disability applications. 

## 2. Muscle Force Transducer and Mechanism

**Figure 1:**
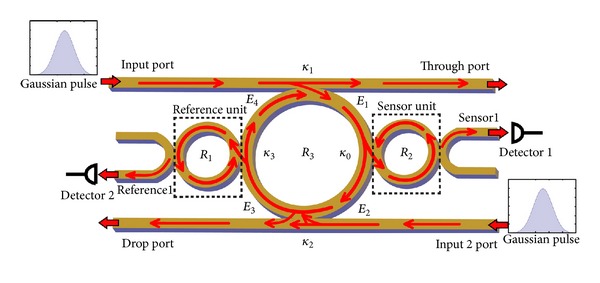
Schematic diagram of a microforce sensing using PANDA ring resonator and deformation shape of sensing ring within the sensing unit with and without external force, where *R*_*s*_ is ring radii, *κ*_*s*_ is coupling constant, and *E*_*s*_ is optical fields.

**Figure 2:**
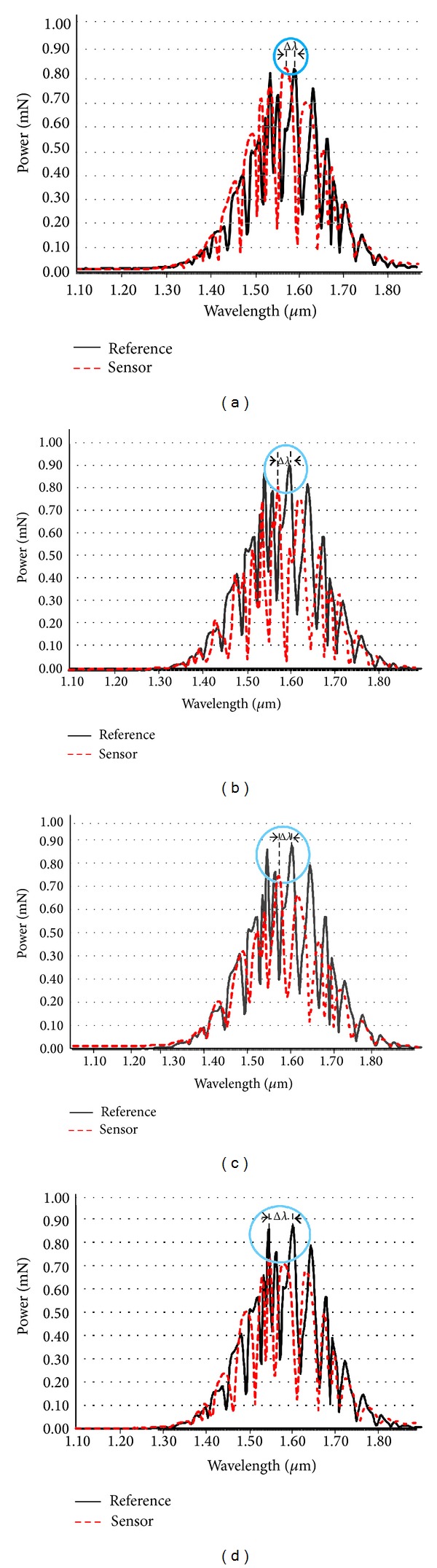
The relationship between interference signals of reference signals (*E*_*R*_1__), with the radius *R*_2_ (black line) = 1.550 *μ*m, and sensing signals (*R*_2_ is varying from 1.65 to 1.95 *μ*m, red line), (a) *R*_2_ = 1.552 *μ*m, (b) *R*_2_ = 1.554 *μ*m, (c) *R*_2_ = 1.556 *μ*m, and (d) *R*_2_ = 1.558 *μ*m.

In this section we attempt to describe the use of optical devices in microscale that can be used to measure the force generated by the movement of the facial muscles, in which those activities can be used to form the relationship between the contraction and relaxation of the facial muscles. The schematic diagram of a sensing transducer using a PANDA ring resonator is as shown in Figure 1, which consists of three microring resonators. The first ring is placed as a reference ring, with radius *R*_1_ = 1.550 *μ*m. The second ring *R*_2_ is the sensing ring, the ring radii are varied from 1.550 to 1.558 *μ*m, and the third ring is used to form the interference signals between signals from the first ring (reference ring) and the second ring (sensing ring), with the radius *R*_3_ = 3.10 *μ*m. In operation, the radius of second ring (sensing ring) is changed by shift of signals circulated in the interferometer ring (*R*_3_). The change in optical path length which is related to the change of the external parameters is compared and measured. The change in optical path *L*(Δ*L*) is affected by the change in ring shape, which is introduced by the strain-optic effects due to the changes in refractive index, *n* by Δ*n*. Finally, the resonant wavelength, *λ*_*m*_, is shifted by Δ*λ*_*m*_ as [(Δ*λ*_*m*_/*λ*_*m*_) = (Δ*n*/*n*)+(Δ*L*/*L*)]. Here, *m* is integer, *n* is the refractive index of the guiding material, and *L* is the circumference of the ring resonator. The movement of muscle on forehead can result in the change in signal output fluctuations (pulses), which can be used to form the pattern recognition for computer commands (codes). In simulation, we assume that the sensing probe (thin film) or other sensing parameters can exert force on the sensing ring (*R*_2_), as shown in Figure 1, whereas the contraction and relaxation of muscles are introduced on the sensing device by means of the elastic modulus of the materials, which caused the difference in the peak spectrum of both signals *Y*_0_ = [(*F*/*A*)/Δ*L*/*L*] = (Stress/Strain) and is described by [29, 30], where the relationship between the force and the change in sensing device length is described by *F* = [(*Y*_0_ *A*_0_)/*L*_0_]Δ*L*. According to the finite difference time domain method (FDTD), the whole system is analyzed, whereas all the parameters are simulated via the computer programming called Optiwave [31]. The simulation results are obtained by using the practical parameters, where a sensing range in terms of wavelength shift (Δ*λ*) with microscale resolution is investigated.  Figure 2 shows results of interference fringes within a PANDA ring wave guide InGaAsP/InP, where the radius *R*_1_ = 1.550 *μ*m, *R*_2_ is varying from 1.552 to 1.558 *μ*m, *R*_3_ = 3.10 *μ*m, *A*_eff_ = 0.3 *μ*m^2^, *n*_eff_ = 3.14, *n*_2_ = 1.3 × 10^−13^ cm^2^/W, *κ*_1_ = *κ*_2_ = 0.5, *γ* = 0.01, and *λ*_0_ = 1.550 nm. The relationship between the varying ring radius *R*_2_ and the wavelength shift (Δ*λ*) is investigated by comparing the sensing and reference signals. The force introduced by the muscle movements causes the changes in optical path lengths, where different fringe patterns can be formed, which is useful for computer interfacing and pattern recognition use. 

## 3. Facial Muscle Sensors and Pattern Recognitions

 The main purpose of this proposal is the use of optical probe to detect the muscle movement, which is induced by forces that occur due to the contraction and relaxation of face muscles, because of the measured expression being associated with the facial gestures, such as closing both eyes, closing right eye, closing left eye, and pulling up the eyebrows, by which we can observe the reaction of the motion or response of the face with these muscle movements, which is generally known as a function of the contraction and relaxation of the facial muscles. By using the received signals, the different signals (interference fringes) and gestures can be used to form the group of commands and used to develop and support the required commands and information via the other electronic devices. In operation, the measurement is performed by using a nonlinear optical ring resonator known as a PANDA ring circuit, which is the right tool. Moreover, it has the ability to perform the measurement due to its small size with its high sensitivity to change the optical frequency/wavelength, where the induced force can occur by the face muscle.

**Figure 3:**
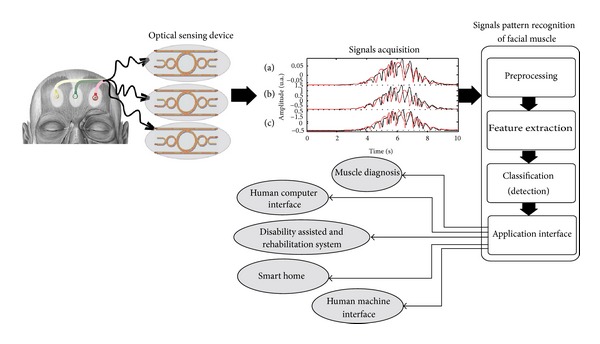
The proposed design system: three probes are connected to human forehead, in which the received signals (a), (b), and (c) are the interference fringes, which can be introduced and detected if the form of optical interference fringe outputs.

Figure 3 shows a block diagram of the proposed system, which consists of four main parts, including optical sensing devices, signal acquisition, signals pattern recognition of facial muscle, and application interface. The optical sensing devices are placed by the thin film optical probes, in which the changes in optical path length introduced by muscle movements can be seen in the forms of interference signals via the signal processing devices, in which the detected signals are then connected to the interfacing devices (application interface), where, finally, the different applications, for instance, muscle diagnosis, smart home, human computer interface, and disability assisted and rehabilitation system can be established. Basically, when the facial muscles are in normal state, the measured value of the signal can be neglected, which is considered as the offset value. In the case of multiple facial gestures, there are three probes that can be used for large areas of sensing distribution, where more accurate results can be used and more applications realized. In this case, the sensor system is implemented as the transmitter of the three probes, each probe can introduce a different pattern, which is determined by the facial gestures from the expression of the face, whereas the signal received from the steps of the sensor system may be a sign that it is not appropriate to use. In practice, the signal acquisition is required to process the signal filtering and form the suitable gesture. In pattern recognition applications, the received signals are required to improve through a process of signal pattern recognition of facial muscle in order to obtain the appropriate pattern, which comprises the substeps including preprocessing to verify the integrity of the signal, feature extraction to obtain a qualified pattern according to the process of classification. Classification or a specific model for the signal, which will lead to the final step is application interface is to take a signal has applications in various fields. 

In case of actual implementation, assuming healthy volunteers were installed optical, devices will be placed according to the position on the left eyebrow, right eyebrow, and above the eyebrows on both sides, as shown in Figure 3. When healthy volunteers have shown facial gestures with behaviors such as closing the right eye, probe will monitor the force of the contraction and relaxation of the facial muscles and makes the received signal different. Therefore, when volunteer closes the right eye, the probe installed above the right eyebrow is higher than other areas due to the contraction of the muscles around the right eyebrow. A probe is located between the eyebrows to show signs of moderate or low level changes; the probe is installed above the left eyebrow to signal a low level change or no changes occur. Handling signals acquisition is carried out through data segmentation, windowing, and the process of signals pattern recognition of facial muscle as means of feature extraction, which are more suitable for training and classification.

In terms of pattern recognitions, the received signals can be used to control and command the computer, in which simple commands without the combination of each probe can be established. In this case, the signals for up to 2^3^ or 8 commands can be formed. By using the same system, a device can be used to assist people with disabilities by setting the different codes and commands, for instance, the use of wheelchair that relies on the movement, such as forward, backward, turn left, turn right, accelerate, and stop which requires 6 commands to control the movement. Finally, we can also use the pattern of different signals from different facial gestures on other benefits such as muscle diagnosis, disability assisted and rehabilitation system, smart home, human computer interface, and human machine interface.

## 4. Conclusion

We have demonstrated the use of a small scale sensor which is formed by a PANDA ring circuit for pattern recognition, computer machine language, and disability applications. In principle, the change in phase of optical device can be configured as the contraction and relaxation of the facial muscles with a PANDA ring resonator. Simulation results of the proposed system are derived from the shift in optical path length of the resonant mode response and the changes in the external environment, that is, stimulated by the optical probe (optical sensing device) behaviors. This sensing probe is small scale size with high sensitivity and good repeatability, which is connected to analytical instruments for data analysis. The received signals from the probe have different wavelengths, which can distinguish the signals with each location of the contraction and relaxation of the facial muscle expression and activity. These signals can be used to form the pattern recognition study and development for the human machine interface and human computer interface applications.
